# Allithiamine Alleviates Hyperglycaemia-Induced Endothelial Dysfunction

**DOI:** 10.3390/nu12061690

**Published:** 2020-06-05

**Authors:** Attila Biró, Arnold Markovics, Mónika Éva Fazekas, Gábor Fidler, Gábor Szalóki, Melinda Paholcsek, János Lukács, László Stündl, Judit Remenyik

**Affiliations:** 1 Institute of Food Technology, Faculty of Agricultural and Food Sciences and Environmental Management, University of Debrecen, H-4032 Debrecen, Hungary; biro.attila@agr.unideb.hu (A.B.); arnoldmarkovich@gmail.com (A.M.); fazekas.monika@agr.unideb.hu (M.É.F.); stundl@agr.unideb.hu (L.S.); 2 Department of Human Genetics, Faculty of Medicine, University of Debrecen, H-4032 Debrecen, Hungary; fidler.gabor@med.unideb.hu (G.F.); paholcsek.melinda@med.unideb.hu (M.P.); 3 1st Department of Pathology and Experimental Cancer Research, Semmelweis University, H-1085 Budapest, Hungary; szaloki.gabor@med.semmelweis-univ.hu; 4 Department of Obstetrics and Gynaecology, University of Debrecen, H-4032 Debrecen, Hungary; lukacs.janos@med.unideb.hu

**Keywords:** allithiamine, garlic, hyperglycaemia, advanced glycation end-products, cytokines

## Abstract

Diabetes mellitus-related morbidity and mortality is a rapidly growing healthcare problem, globally. Several nutraceuticals exhibit potency to target the pathogenesis of diabetes mellitus. The antidiabetic effects of compounds of garlic have been extensively studied, however, limited data are available on the biological effects of a certain garlic component, allithiamine. In this study, allithiamine was tested using human umbilical cord vein endothelial cells (HUVECs) as a hyperglycaemic model. HUVECs were isolated by enzymatic digestion and characterized by flow cytometric analysis using antibodies against specific marker proteins including CD31, CD45, CD54, and CD106. The non-cytotoxic concentration of allithiamine was determined based on MTT, apoptosis, and necrosis assays. Subsequently, cells were divided into three groups: incubating with M199 medium as the control; or with 30 mMol/L glucose; or with 30 mMol/L glucose plus allithiamine. The effect of allithiamine on the levels of advanced glycation end-products (AGEs), activation of NF-κB, release of pro-inflammatory cytokines including IL-6, IL-8, and TNF-α, and H_2_O_2_-induced oxidative stress was investigated. We found that in the hyperglycaemia-induced increase in the level of AGEs, pro-inflammatory changes were significantly suppressed by allithiamine. However, allithiamine could not enhance the activity of transketolase, but it exerts a potent antioxidant effect. Collectively, our data suggest that allithiamine could alleviate the hyperglycaemia-induced endothelial dysfunction due to its potent antioxidant and anti-inflammatory effect by a mechanism unrelated to the transketolase activity.

## 1. Introduction

In recent decades, several research have focused on the pharmacologically active, plant-derived compounds [1]. Extensive research on physiological effects of nutraceuticals is expected to continue in the near future. A recently published review unequivocally declared that the ongoing discovery of naturally occurring drugs stands as a major contributor to cope with diseases, reaching high prevalence, globally [2].

Among plants having significant pharmacological activity, garlic (*Allium sativum* L.) is among the most studied ones [3]. Several studies have shown that garlic exerts antioxidant, antimicrobial [4], anti-inflammatory, immunomodulatory [5], antithrombotic [6], anti-atherosclerotic [7], antihypertensive [8], and anti-carcinogenic [9] effects. The biological effects of garlic are mainly attributed to its characteristic organosulfur compounds, including alliin, allicin, ajoene, S-allylmercaptocystein, diallyl disulfide, and S-allyl-cysteine, among others. [10]. Limited data in the scientific literature are available on the biological effects of another garlic component, allithiamine, which is a less polar thiamine (B_1_-vitamin) derivative and, similar to the molecules mentioned above, has a prop-2-en-1-yl disulfanyl moiety. According to a recent study, allithiamine is also accumulated in red sweet pepper (*Capsicum annuum* L.) seeds, implying that its occurrence is more frequent than as thought until now. Nevertheless, several studies revealed that numerous garlic compounds have beneficial effects on hyperglycaemia in diabetes mellitus [11].

Diabetes mellitus is a rapidly growing public health burden, particularly in developed countries [12]. Diabetes mellitus is a metabolic, endocrine disorder, which can cause an acute life-threatening homeostasis imbalance as well as chronically developing micro- and macrovascular complications (blindness, neuropathy, myocardial infarction, stroke, etc.) [13]. There is a common agreement that endothelial dysfunction precedes the development of micro- and macrovascular complications associated with diabetes mellitus [14]. These complications are caused—at least partially—by the detrimental effects of hyperglycaemia, which affects endothelial cell biology by accelerating the formation of advanced glycation end-products (AGEs), thereby increasing pro-inflammatory signaling and resulting in oxidative stress [15].

Glucose reacts with an amino group of the circulating proteins during the formatting of AGEs. The level of AGEs elevates heavily in the presence of chronic hyperglycaemia to evoke both damaging biological functions of glycated molecules, resulting in altered intracellular signaling, gene expression, release of pro-inflammatory molecules, and enhanced oxidative stress by bonding to cell surface receptors (RAGE), and so consequently, AGEs play a major role in diabetic microvascular complications [16]. Hyperglycaemia, alone can trigger inflammation by activating the pro-inflammatory transcription factor nuclear κB (NF-κB), resulting in an increased inflammatory chemokine and cytokine release including interleukin-6 (IL-6), interleukin-8 (IL-8), and tumor necrosis factor-α (TNF-α), among others. [17]. A recent study reported that alleviating the release of pro-inflammatory cytokines has a beneficial effect in chronic hyperglycaemia [18]. In addition, a high level of glucose enhances oxidative stress, when the rate of oxidant production exceeds the rate of oxidant scavenging [19]. In the case of hyperglycaemia, there are both enhanced oxidant production and impaired antioxidant defenses by multiple interacting pathways [20]. Studies have demonstrated that compounds with a strong antioxidant property can potentially be effective in delaying diabetes-related complications.

To date, there is no preclinical evidence for the antidiabetic effect of allithiamine, therefore, the main objective of our current research was to study whether this compound is able to exert a beneficial effect on diabetes. Primary cultured human umbilical cord vein endothelial cells (HUVECs) were used as a unique hyperglycaemic model, which appeared to be ideally capable to investigate the level of AGEs, antioxidant status, and pro-inflammatory cytokines.

## 2. Materials and Methods

### 2.1. Materials

#### Chemicals

All reagents were obtained from the distributor of iBioTech Hungary Ltd. (Budapest, Hungary) and DIAGON Ltd. Hungary (Budapest, Hungary).

### 2.2. Methods

#### 2.2.1. Preparation and Purification of Allithiamine

Preparation and purification of allithiamine were carried out based on the method of our recent allithiamine-oriented study [21]. Briefly, allyl thiosulphate and thiamine hydrochloride with an opening thiazole ring were reacted. As a result of the reaction, many organosulfur compounds were formed, including allithiamine. Reaction products were separated and allithiamine was purified by reversed-phase chromatography using LaChrom HPLC equipped with a diode array detector. (Hitachi, Osaka, Japan). To confirm the accuracy and efficiency of the allithiamine synthesis and purification, matrix-assisted laser desorption/ionization mass spectrometric (MALDI-MS) analysis and HPLC-MS/MS fragmentation were performed applying a Bruker Biflex MALDI-TOF mass spectrometer (Bruker, Billerica, MA, USA) and Thermo Scientific Q Exactive Orbitrap mass spectrometer (Thermo Fisher Scientific Inc., Waltham, MA, USA).

#### 2.2.2. Isolation and Treatment of Primary HUVECs

The HUVECs were isolated from human umbilical cords and maintained according to the method previously described [22]. In our experiments, supplemented M199 medium was used as a control and had 5.6 mMol/L glucose. To create a hyperglycaemic model, glucose was added to M199 for a final concentration of 30 mMol/L.

#### 2.2.3. Characterization of HUVECs by Flow Cytometry

HUVECs were incubated with four fluorescent dye-labeled antibodies, involving fluorescein-isothiocyanate (FITC)-labeled mouse anti-human CD31, phycoerythrin (PE)-labeled anti-human CD54, allophycocyanin (APC)-labeled mouse anti-human CD106, and PerCP-Cy5.5-labeled mouse anti-human CD45 (BD Biosciences, Franklin Lakes, NJ, USA), and then characterized by using a FACSAriaIII Cell Sorter (Becton Dickinson, Mountain View, CA, USA) [22].

#### 2.2.4. Determination of Cellular Viability

Cell viability was determined by an MTT (3-[4,5-dimethylthiazol-2-yl]-2,5 diphenyltetrazolium bromide) assay. Cells were plated into 96-well plates at a density of 20,000 cells per well in quadruplicates and treated with allithiamine of different concentrations (0.1, 0.5, 1, 5, 10, and 50 μg/mL) and without allithiamine (control group) for 24, 48, and 72 h. The medium was removed and incubated with 100 μL of MTT solution (0.5 mg/ml dissolved in Dulbecco’s modified Eagle’s medium) for 3 h. Subsequently, the formazan crystals were dissolved in 100 μL solubilizing solution (81% (*v*/*v*) isopropyl alcohol, 9% (*v*/*v*), 1 M HCl, 10% (*v*/*v*) Triton X-100) and the absorbance was measured at 465 nm by using a Clariostar microplate reader (BMG Labtech, Ortenberg, Germany). Cell viability at different allithiamine concentrations was expressed relative to 100% of the control group.

#### 2.2.5. Determination of Apoptosis

The mitochondrial membrane potential of the HUVECs was determined by using 1,1′,3,3,3′,3′-hexamethylindodicarbocyanin iodide (DilC_1_(5)) dye. The decrease in the DilC_1_(5) intensity indicated mitochondrial depolarization, i.e., the onset of early apoptotic processes of HUVECs [23,24]. Cells were seeded to 96-well plates at a density of 20,000 cells per well treated with allithiamine of different concentrations (0.1, 0.5, 1, 5, 10, and 50 μg/mL). After the removal of the medium, the cells were incubated for 30 min with 50 μL/well DilC_1_(5) working solution (50 nM dissolved in Dulbecco’s modified Eagle’s medium). After incubation, cells were washed twice with PBS and the fluorescence of DilC_1_(5) was measured at 630 nm excitation and 670 nm emission wavelengths by using a Clariostar microplate reader (BMG Labtech, Ortenberg, Germany). The results were expressed relative to 100% of the control group.

#### 2.2.6. Determination of Necrosis

Necrotic processes were evaluated by SYTOX Green staining. The dye is able to penetrate only necrotic cells with ruptured plasma membranes and then binds to the nucleic acids, whereas intact cells with unimpaired surface membranes show a negligible SYTOX Green staining intensity [23,24]. Cells were cultured in 96-well plates, and treated as indicated in Section 2.2.5. After the removal of medium, cells were incubated for 30 min with 50 μL/well SYTOX Green dye (1 μM dissolved in Dulbecco’s modified Eagle’s medium) and then washed with PBS. The fluorescence of SYTOX Green was measured at 490 nm excitation and 520 nm emission wavelengths by using a Clariostar microplate reader (BMG Labtech, Ortenberg, Germany). The results were expressed relative to 100% of the control group.

#### 2.2.7. Performing ELISAs

##### Measurement of Advanced Glycation End-Products

The assay was performed according to the manufacturer’ instructions by using an OxisSelect^TM^ Advanced Glycation End Product (AGE) Competitive ELISA Kit (Cell Biolabs Inc., San Diego, CA, USA).

##### Measurement of NF-κB

The assay was performed according to the manufacturer’ instructions by using a Human NF-κB p65 Sandwich ELISA Kit (Fine Biological Technology Ltd., Wuhan, China)

#### 2.2.8. Determination of Cytokine Release

HUVECs were seeded into a 6-well plate (500,000 cells/well), and were incubated in 5 mMol/L glucose and 30 mMol/L glucose with or without 5 μg/mL allithiamine for 6, 12, or 24 h. Supernatants were collected, centrifuged for 10 min 10,000 r·min^−1^ and the released amount of IL-6, IL-8, and TNF-α was determined by using a MILLIPLEX MAP Human cytokine/chemokine Magnetic Bead Panel (EMD Millipore Corp., Billerica, MA, USA) based on the manufacturer’s recommendation.

#### 2.2.9. Measurement of Transketolase Activity

Transketolase activity was measured by adding 100 μL cytosolic fraction to 200 μL reaction mixture containing 14.8 mMol/L ribose-5-phosphate, 253 μMol/L NADH, 185 U/mL triosephosphate isomerase, and 21.5 U/mL glycerol-3-phosphate dehydrogenase in Tris buffer (pH = 7.9). The optical density was measured at 340 nm immediately and then every 10 min for 2 h by using a Clariostar microplate reader (BMG Labtech, Ortenberg, Germany). The activity was calculated from the difference in the optical density readings at 10 and 80 by min using the extinction coefficient of NADH. Results are expressed in nMol/min/mg protein.

#### 2.2.10. Determination of Protein Content

The protein concentrations were determined in the cell lysate by using a Pierce BCA Protein assay (Pierce Biotechnology Inc., Rockford, IL, USA). Protease inhibitor cocktail (Pierce Biotechnology Inc., Rockford, IL, USA) was added to the cell lysate prior to its storage or measurement.

#### 2.2.11. Determination of ROS Production

The cells were seeded in a 24-well plate and exposed to 100 μMol/L 2′,7′-dichlorofluorescin diacetate (DCFDA) for 1 h at 37 °C to label the intracellular ROS. After incubation, the cells were washed twice with PBS and divided into three groups: incubating with M199 medium as control; or with 100 μMol/L H_2_O_2_; or with 100 μMol/L H_2_O_2_ plus 5 μg/mL allithiamine. Fluorescence (excitation = 485 nm; emission = 530 nm) was assessed by using a Clariostar microplate reader (BMG Labtech, Ortenberg, Germany).

#### 2.2.12. Statistical Analysis

For multiple comparisons, results were analyzed by an ANOVA followed by a modified *t*-test for repeated measures according to Bonferroni’s method. Data were presented as mean ± SEM. Differences were considered statistically significant, when *p* < 0.05.

### 2.3. Ethics

The study was conducted in accordance with the Declaration of Helsinki, and the protocol was approved by the Ethics Committee of the University of Debrecen (registration number RKEB/IKEB 3712-2012).

## 3. Results

### 3.1. Purification and Verification of Allithiamine

Since allithiamine is not commercially available and that isolation from the plant does not work in large amounts, chemically prepared and purified allithiamine was applied for our experiments. The synthesis of allithiamine resulted in a wide variety of organosulfur compounds, which were separated by using the reversed-phase chromatographic method. After the chromatographic separation and purification of allithiamine, a fraction of allithiamine was verified by using the MALDI-MS and HPLC-MS/MS techniques. These methods clearly indicated that the chromatographic purification of allithiamine was accurate and efficient. The chromatogram (Supplementary Figure S1) of the synthetic mixture and MALDI-MS spectrum (Supplementary Figure S2) as well as the MS^2^ spectrum (Supplementary Figure S3) of purified allithiamine can be seen in the Supplemental Materials.

### 3.2. Flow Cytometric Studies

**Figure 1:**
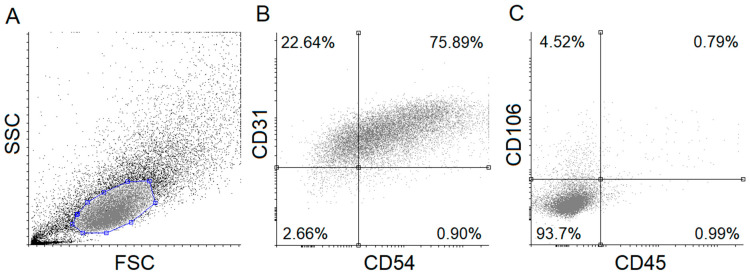
Flow cytometric analysis of human umbilical cord vein endothelial cells (HUVECs). Isolated HUVECs were verified using specific fluorescent-labeled antibodies. Forward- and side-scatter plot and dot-plots (**A**) of HUVEC positive (CD54, CD31) (**B**) and negative (CD45, CD106) (**C**) markers are shown. FSC: Forward scatter, SSC: Side scatter.

Isolated HUVECs were characterized to positive and negative marker expressions by applying flow cytometry and antibodies against specific marker proteins, a routinely used method in our recent HUVEC-oriented study [22]. CD106, CD45, CD31, and CD54 were applied to label the cells. As shown in Figure 1, the isolated HUVECs showed a high CD31 and CD54 positive marker expression, while approximately 93% of the cells did not express CD45 and CD106 (negative markers). These results suggest that the isolation of HUVECs was sufficiently accurate and efficient.

### 3.3. Determination of Optimal Concentration of Allithiamine

#### Up to 5 μg/mL, Allithiamine Treatment Has No Effect on Survival Rate of HUVECs

**Figure 2:**
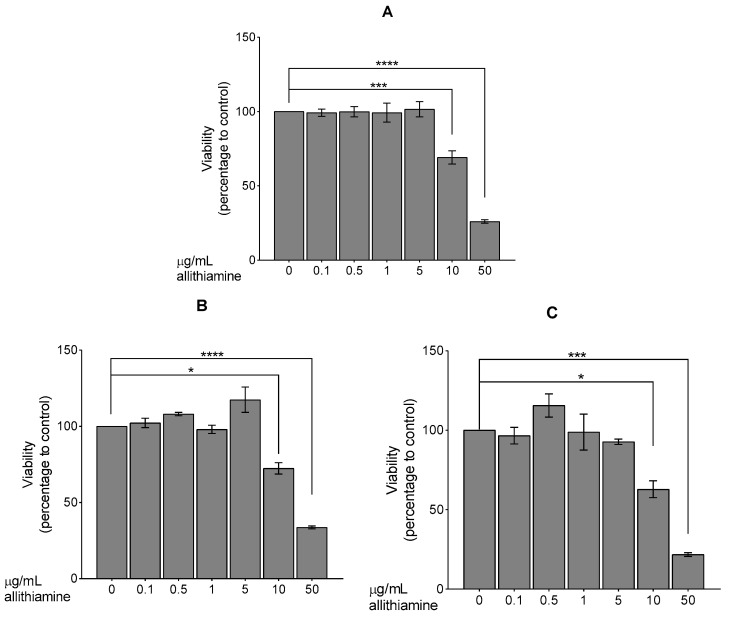
Viability of HUVECs was examined after 24 (**A**), 48 (**B**), or 72 (**C**) h. Results are expressed in the percentage of the control (0 μg/mL allithiamine). Data are expressed as the mean ± SEM of three individual experiments. Two additional experiments yielded similar results. *, ***, and **** mark significant (*p* < 0.05, 0.0005, and 0.0001, respectively) differences compared with control.

At first, the effect of allithiamine on cell viability was evaluated by using an MTT assay. HUVECs were exposed to allithiamine at different concentrations (0.1–50 μg/mL) for 24, 48, and 72 h. We found that up to 5 μg/mL allithiamine did not decrease the viability of HUVECs in this time window (Figure 2).

**Figure 3:**
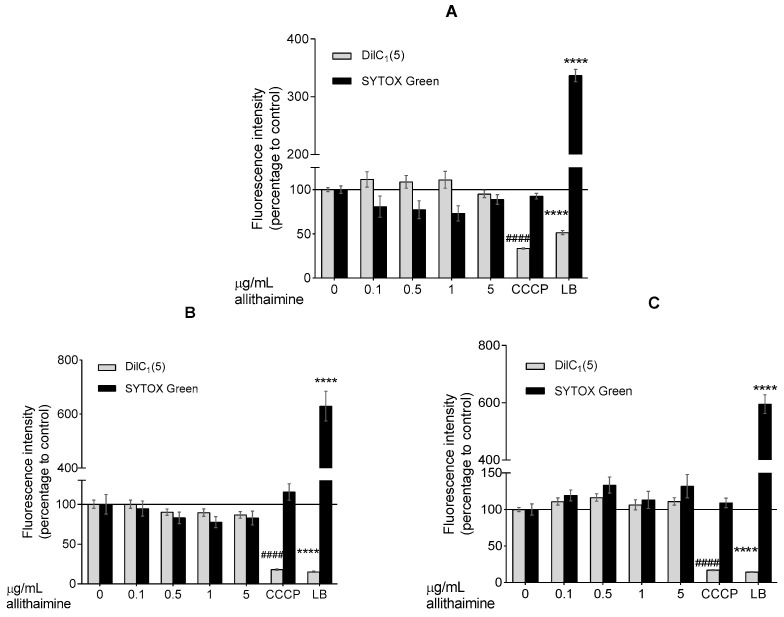
Fluorescent DilC_1_(5) and SYTOX Green labeling. Effect of allithiamine on apoptosis and necrosis after 24 (**A**), 48 (**B**), or 72 (**C**) h. Results (intensity of fluorescence) are expressed in the percentage of the control (0 μg/mL allithiamine; 100% is represented by the solid lines). Apoptosis is indicated by a decrease in fluorescence of DilC_1_(5), and necrosis is indicated by an increase in fluorescence of SYTOX Green. Data are expressed as the mean ± SEM of three individual experiments. Two additional experiments yielded similar results. **** and #### mark significant (*p* < 0.0001 in both cases) differences compared with the control group (0 μg/mL allithiamine). CCCP: carbonyl cyanide m-chlorophenyl hydrazone (positive control for apoptosis); LB: lysis buffer (positive control for necrosis). SYTOX Green: non-permeable fluorescent nucleic acid dye; DilC_1_(5): 1,1′,3,3,3′,3′-hexamethylindodicarbocyanin iodide dye.

In order to exclude the possibility of early apoptotic and necrotic events, which are not obvious alterations in the MTT assay, we further evaluated the effect of allithiamine whilst performing fluorescent labeling (DilC_1_(5) and SYTOX Green dyes). The results show that allithiamine, in line with the MTT data, did not induce a necrotic and apoptotic process and can be used without the risk of any biologically relevant cytotoxic actions in this concentration range (≤5 μg/mL; 24–72 h; Figure 3). Based on these preliminary experiments, a concentration of 5 μg/mL allithiamine was selected for further investigations.

### 3.4. Allithiamine Can Reduce the Level of Advanced Glycation End-Products (AGEs)

**Figure 4:**
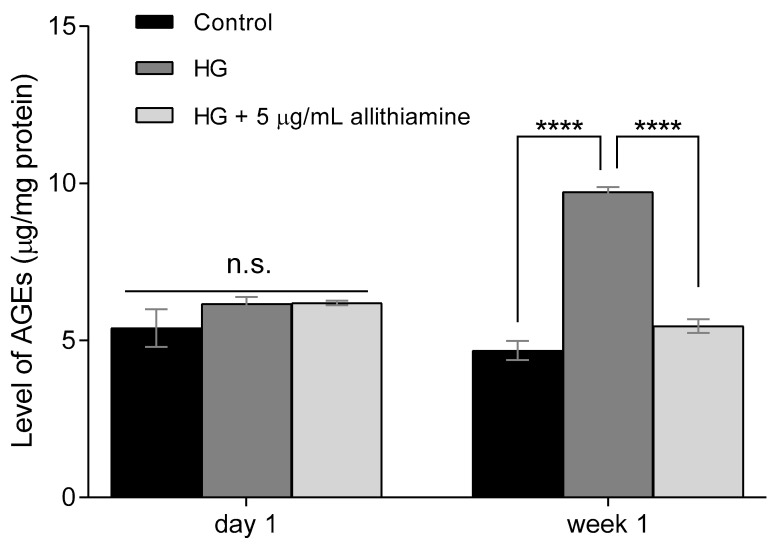
Effect of allithiamine on level of advanced glycation end-products (AGEs) in HUVECs after one day and one week. Data are expressed as the mean ± SEM of three individual experiments. **** marks a significant (*p* < 0.0001) difference between the control and HG, and between HG and HG+5 μg/mL allithiamine after one week. n.s.: not significant. HG: hyperglycaemia (30 mMol/L glucose).

In cases of chronic hyperglycaemia, cells are exposed to prolonged elevated glucose concentrations leading to an excessive formation of AGEs [25]. Several studies have reported the positive effect of garlic sulfur compounds on the level of AGEs [26,27]. Therefore, we aimed to study the effects of allithiamine on the level of AGEs after one-day and one-week exposure to hyperglycaemia. As expected, 30 mMol/L glucose significantly increased the level of AGEs in HUVECs (Figure 4). We found that this hyperglycaemia-induced nearly 2-fold increase was significantly suppressed by the above-revealed non-cytotoxic concentration (5 μg/mL) of allithiamine.

### 3.5. Allithiamine Can Alleviate the Hyperglycaemia-Induced Inflammatory Response in HUVECs

**Figure 5:**
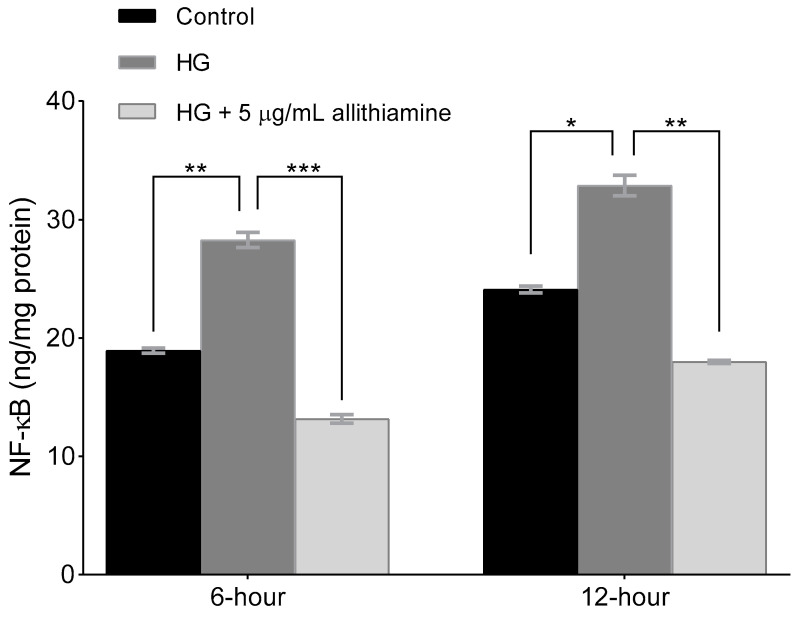
Effect of allithiamine on the activation of NF-κB in HUVECs after 6 and 12 h. Data are expressed as the mean ± SEM of three individual experiments *, **, and *** mark significant (*p* < 0.05, *p* < 0.005, and *p* < 0.0005) differences between the control and HG, and between HG and HG+5 μg/mL allithiamine, HG: hyperglycaemia (30 mMol/L glucose).

**Figure 6:**
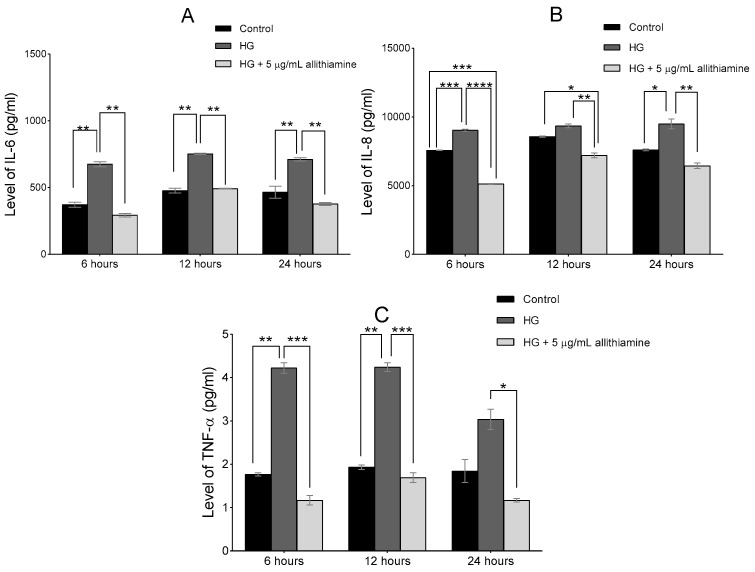
Effect of allithiamine on the level of pro-inflammatory cytokines including IL-6 (**A**), IL-8 (**B**), and TNF-α (**C**) in HUVECs after 6, 12, and 24 h. Data are expressed as the mean ± SEM of three individual experiments *, **, ***, and **** mark significant (*p* < 0.05, *p* < 0.005, *p* < 0.0005, and *p* < 0.0001) differences between the control and HG, between HG and HG+5 μg/mL allithiamine, and between control and HG+5 μg/mL allithiamine. HG: hyperglycaemia (30 mMol/L glucose).

Several studies reported the close relationship between hyperglycaemia and the inflammatory response of endothelium. In order to get a deeper insight into the effects of allithiamine on these inflammatory reactions, we assessed the activation of NF-κB in the cell lysate after 6 and 12 h and the release of TNF-α, IL-6, and IL-8 in the cell supernatant after 6, 12, and 24 h incubation in 5 mMol/L glucose and 30 mMol/L glucose with or without 5 μg/mL allithiamine. We found that the hyperglycaemic condition was able to trigger the inflammatory processes at an early time. The activation of NF-κB was significantly increased after the hyperglycaemic treatments in all the sampling times. Allithiamine was able to decrease this increment (Figure 5). The secretion of IL-6 and IL-8 was significantly increased after the hyperglycaemic treatments in all the sampling times. The TNF-α secretion significantly increased after the hyperglycaemic treatment at 6 and 12 h. At 24 h, a statistically not significant but marked biological change was observed. We found that allithiamine could alleviate the hyperglycaemia-induced inflammatory response by suppressing the pro-inflammatory cytokines mentioned above (Figure 6).

### 3.6. Allithiamine Has No Effect on Transketolase Activity

**Figure 7:**
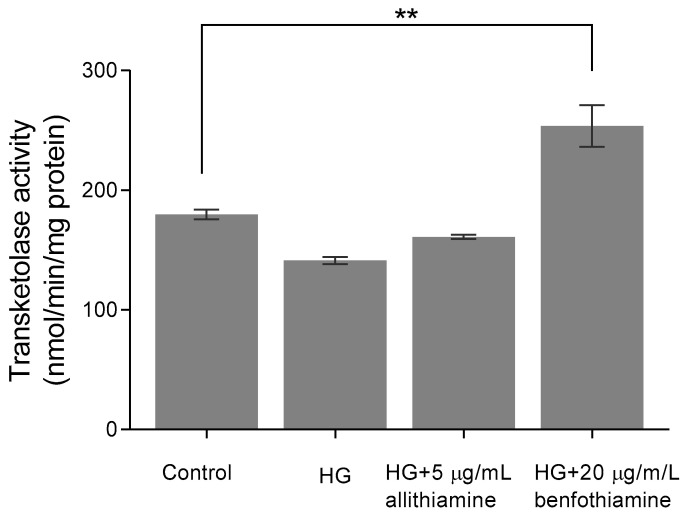
Effect of allithiamine on the transketolase activity in HUVECs after 6 h of incubation. Data are expressed as the mean ± SEM of three individual experiments. Two additional experiments yielded similar results. ** marks a significant (*p* < 0.005) difference between the control and HG+20 μg/mL benfotiamin. HG: hyperglycaemia (30 mMol/L glucose).

Considering the encouraging results presented above, we assayed to determine which mechanism may be responsible for the positive biological changes. First, we assumed that allithiamine, similar to thiamine derivatives, is able to enhance the activity of transketolase, a key enzyme in the pentose phosphate pathway. Benfothiamine is a potent transketolase activator [28], therefore, we applied it as a positive control in our experiments involving transketolase. The enzyme activity was evaluated in HUVECs after 6, 12, and 24 h of incubation. In the 6-h sample, we observed that 20 μg/mL benfothiamine increased significantly the transketolase activity in cells incubated in 30 mM glucose compared with cells incubated in 5 mM glucose and 30 mM glucose. However, 5 μg/mL allithiamine in cells incubated in 30 mM glucose did not change the transketolase activity significantly (Figure 7). Further incubations did not result in a significant increase in the transketolase activity (data not shown).

### 3.7. Allithiamine Exerts a Potent Antioxidant Effect

**Figure 8:**
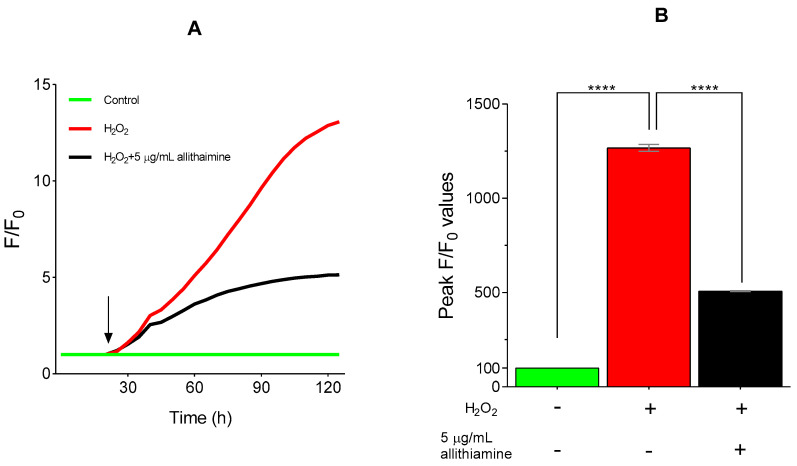
Antioxidative effect of allithiamine in HUVECs incubated with or without 100 μMol/L H_2_O_2_. Fluorescent intensity was normalized to the baseline (**A**). H_2_O_2_ was administrated as indicated by the arrow. Statistical analysis was performed at the peak fluorescence (F/F_0_) values (**B**). Data are expressed as the mean ± SEM of three individual experiments. Two additional experiments yielded similar results. **** marks a significant (*p* < 0.0001) difference between the control and H_2_O_2_, and between H_2_O_2_ and H_2_O_2_+5 μg/mL allithiamine. H_2_O_2_: hydrogen-peroxide. – indicates the absence of treatment substance; + indicates the presence of treatment substance.

As small molecule organosulfur compounds with a prop-2-en-1-yl disulfanyl moiety of garlic have strong antioxidant properties [29], we further intended to investigate the potential antioxidant effect of allithiamine. To assess the antioxidant capacity of allithiamine, hydrogen-peroxide (H_2_O_2_), a routinely used oxidative agent [30], was applied in our HUVEC model, ensuring an enhanced ROS production and imbalance in the oxidative status of cells. As expected, the administration of H_2_O_2_ significantly increased the production of ROS. Allithiamine was able to eliminate a significant part of this increment, indicating the potent antioxidant effect of allithiamine (Figure 8).

## 4. Discussion

In recent years, several research indicate that plant-derived compounds will be among the most important sources of new drugs [31]. Plants tend to produce several chemically highly diverse secondary metabolites, which may be suitable for exerting positive effects in human diseases [32]. Numerous studies have shown that garlic compounds (ajoene, alliin, allicin, diallyl disulfide S-allyl-cystein, etc.) are particularly valuable in this regard [33]. An experiment with mice demonstrated that hyperglycaemia was suppressed by ajoene treatment [34]. Another study revealed that allicin had a protective effect on hyperglycaemia-induced injury in aortic endothelial cells [35]. Findings of a study in rats suggest that S-allyl-Cystein treatment exerts a therapeutic protective effect on diabetes by decreasing oxidative stress [36]. Furthermore, comprehensive studies on garlic showed its therapeutic potential in various diseases accompanied by hyperglycaemia, including diabetes mellitus [37]. Our objective was to investigate the effect of allithiamine, a less-studied garlic component, on hyperglycaemia-induced endothelial pathologic changes (AGEs yielding, inflammatory processes, ROS production) in a HUVEC model.

Primarily, HUVECs were characterized by flow cytometric analysis using antibodies against specific marker proteins including CD31, CD45, CD54, and CD106. The results proved that the cells used in our experiments were endothelial cells, indeed. Subsequently, the non-cytotoxic concentration of allithiamine was determined by using an MTT assay and fluorescent labeling with DilC_1_(5) (examination of apoptosis) and SYTOX Green (investigation of necrosis) dyes. The viability tests of HUVECs after exposure to allithiamine at different concentrations (0.1–5 µg/mL) for up to 72 h indicate that cells were not significantly affected.

In parallel with the research on the garlic component which showed a suppressed formation of AGEs [27], excellent markers of tissue damage caused by persistent hyperglycaemia [38], we examined the effect of allithiamine on the level of AGEs. We found that a prolonged (one week) hyperglycaemia-induced increase in the level of AGEs was significantly suppressed by allithiamine.

In order to investigate the effects of allithiamine on inflammatory processes, we examined NF-κB which has been proven to be upregulated under hyperglycaemic conditions. NF-κB has a pivotal role in the inflammatory process as a transcriptional factor of several pro-inflammatory cytokines [39]. We found that allithiamine alleviated the expression of NF-κB caused by an elevated glucose level. We also assessed the release of several cytokines including IL-6, IL-8, and TNF-α, which faithfully reflect the inflammatory state of HUVECs [40]. To observe the early release of the preformed cytokine pool and secretion of de novo-synthetized cytokines, we measured the level of the abovementioned molecules after 6, 12, and 24 h of treatment. We also revealed that allithiamine was able to significantly decrease the level of the investigated cytokine at the time of sampling.

Seeking possible reasons behind the positive effect of allithiamine, two plausible explanations were raised. At first, we assumed that allithiamine is able to enhance the transketolase activity, resulting in a decreased flux of hyperglycaemia-induced pathologic pathways involving the polyol, AGEs, PKC, and hexosamine pathways, similar to thiamine [41] and benfothiamine [28]. However, we found that 5 μg/mL allithiamine did not change the transketolase activity significantly. We assumed that this non-toxic concentration may be rather negligible to increase the transketolase activity. At second, the widely researched garlic compounds having prop-2-en-1-yl moiety, as a two-edged sword, are able to attenuate the oxidative damage [29] and exert a beneficial anti-inflammatory effects as a slow H_2_S donor [42,43]. Allithiamine possesses a prop-2-en-1-yl moiety, as well. In order to get a deeper insight into the putative antioxidant effect of allithiamine, an experiment was performed to verify the eliminating ability of ROS using H_2_O_2_ as an oxidative agent. Allithiamine was able to eliminate the significant part of ROS after the administration of H_2_O_2_. Consequently, our results clearly indicate that allithiamine exhibits a potent antioxidant effect, which may be responsible for the improvement in hyperglycaemia-induced endothelial dysfunction.

## 5. Conclusions

Collectively, in this study, we observed that—without influencing the viability, necrosis, or apoptosis of HUVECs—allithiamine was able to attenuate particular negative effects of an elevated glucose level by its potent antioxidant effect and a mechanism unrelated to the transketolase activity. Further studies are needed to elucidate the mechanisms of action of allithiamine as well as its supposed beneficial role in the spectrum of plant-derived bioactive molecules. However, our results contribute to a better understanding of this relatively less-researched compound, with particular respect to its beneficial effects on hyperglycaemia.

## Supplementary Materials

The following are available online at https://www.mdpi.com/2072-6643/12/6/1690/s1, Figure S1: HPLC chromatogram of allithiamine at 250 nm, Figure S2: MALDI-TOF spectrum of purified allithiamine, Figure S3: MS^2^ spectrum of allithiamine.

## References

[1] Dias D.A., Urban S., Roessner U. (2012). A historical overview of natural products in drug discovery. Metabolites.

[2] Thomford N.E., Senthebane D.A., Rowe A., Munro D., Seele P., Maroyi A., Dzobo K. (2018). Natural Products for Drug Discovery in the 21st Century: Innovations for Novel Drug Discovery. Int. J. Mol. Sci..

[3] Bayan L., Koulivand P.H., Gorji A. (2014). Garlic: A review of potential therapeutic effects. Avicenna J. Phytomed..

[4] Jang H.-J., Lee H.-J., Yoon D.-K., Ji D.-S., Kim J.-H., Lee C.-H. (2017). Antioxidant and antimicrobial activities of fresh garlic and aged garlic by-products extracted with different solvents. Food Sci. Biotechnol..

[5] Arreola R., Quintero-Fabián S., López-Roa R.I., Flores-Gutiérrez E.O., Reyes-Grajeda J.P., Carrera-Quintanar L., Ortuño-Sahagún D. (2015). Immunomodulation and anti-inflammatory effects of garlic compounds. J. Immunol. Res..

[6] Ariga T., Seki T. (2006). Antithrombotic and anticancer effects of garlic-derived sulfur compounds: A review. BioFactors.

[7] Sobenin I.A., Andrianova I.V., Lakunin K.Y., Karagodin V.P., Bobryshev Y.V., Orekhov A.N. (2016). Anti-atherosclerotic effects of garlic preparation in freeze injury model of atherosclerosis in cholesterol-fed rabbits. Phytomed. Int. J. Phytother. Phytopharmacol..

[8] Ried K., Fakler P. (2014). Potential of garlic (*Allium sativum*) in lowering high blood pressure: Mechanisms of action and clinical relevance. Integr. Blood Press Control.

[9] Dorant E., van den Brandt P.A., Goldbohm R.A., Hermus R.J., Sturmans F. (1993). Garlic and its significance for the prevention of cancer in humans: A critical view. Br. J. Cancer.

[10] Omar S.H., Al-Wabel N.A. (2010). Organosulfur compounds and possible mechanism of garlic in cancer. Saudi Pharm. J..

[11] Wang J., Zhang X., Lan H., Wang W. (2017). Effect of garlic supplement in the management of type 2 diabetes mellitus (T2DM): A meta-analysis of randomized controlled trials. Food Nutr. Res..

[12] Animaw W., Seyoum Y. (2017). Increasing prevalence of diabetes mellitus in a developing country and its related factors. PLoS ONE.

[13] Fowler M.J. (2008). Microvascular and Macrovascular Complications of Diabetes. Clin. Diabetes.

[14] Domingueti C.P., Dusse L.M.S.A., Carvalho M.d.G., de Sousa L.P., Gomes K.B., Fernandes A.P. (2016). Diabetes mellitus: The linkage between oxidative stress, inflammation, hypercoagulability and vascular complications. J. Diabete Complicat..

[15] Ren X., Ren L., Wei Q., Shao H., Chen L., Liu N. (2017). Advanced glycation end-products decreases expression of endothelial nitric oxide synthase through oxidative stress in human coronary artery endothelial cells. Cardiovasc. Diabetol..

[16] Rhee S.Y., Kim Y.S. (2018). The Role of Advanced Glycation End Products in Diabetic Vascular Complications. Diabetes Metab. J..

[17] Suryavanshi S.V., Kulkarni Y.A. (2017). NF-κβ: A Potential Target in the Management of Vascular Complications of Diabetes. Front. Pharmacol..

[18] Sun Q., Li J., Gao F. (2014). New insights into insulin: The anti-inflammatory effect and its clinical relevance. World J. Diabetes.

[19] Birben E., Sahiner U.M., Sackesen C., Erzurum S., Kalayci O. (2012). Oxidative stress and antioxidant defense. World Allergy Organ. J..

[20] Matough F.A., Budin S.B., Hamid Z.A., Alwahaibi N., Mohamed J. (2012). The role of oxidative stress and antioxidants in diabetic complications. Sultan Qaboos Univ. Med. J..

[21] Biro A., Gál F., Hegedűs C., Batta G., Cziáky Z., Peitl B., Stündl L., Gyémánt G., Remenyik J. (2018). Isolation of allithiamine from Hungarian red sweet pepper seed (*Capsicum annuum* L.). Heliyon.

[22] Biro A., Markovich A., Homoki J.R., Szőllősi E., Hegedűs C., Tarapcsák S., Lukács J., Stündl L., Remenyik J. (2019). Anthocyanin-Rich Sour Cherry Extract Attenuates the Lipopolysaccharide-Induced Endothelial Inflammatory Response. Molecules.

[23] Markovics A., Tóth K.F. (2019). Nicotinic acid suppresses sebaceous lipogenesis of human sebocytes via activating hydroxycarboxylic acid receptor 2 (HCA(2)). J. Cell. Mol. Med..

[24] Oláh A., Markovics A., Szabó-Papp J., Szabó P.T., Stott C., Zouboulis C.C., Bíró T. (2016). Differential effectiveness of selected non-psychotropic phytocannabinoids on human sebocyte functions implicates their introduction in dry/seborrhoeic skin and acne treatment. Exp. Dermatol..

[25] Huebschmann A.G., Regensteiner J.G., Vlassara H., Reusch J.E. (2006). Diabetes and advanced glycoxidation end products. Diabetes Care.

[26] Elosta A., Slevin M., Rahman K., Ahmed N. (2017). Aged garlic has more potent antiglycation and antioxidant properties compared to fresh garlic extract in vitro. Sci. Rep..

[27] Ahmad M.S., Pischetsrieder M., Ahmed N. (2007). Aged garlic extract and S-allyl cysteine prevent formation of advanced glycation endproducts. Eur. J. Pharmacol..

[28] Hammes H.P., Du X., Edelstein D., Taguchi T., Matsumura T., Ju Q., Lin J., Bierhaus A., Nawroth P., Hannak D. (2003). Benfotiamine blocks three major pathways of hyperglycemic damage and prevents experimental diabetic retinopathy. Nat. Med..

[29] Chung L.Y. (2006). The antioxidant properties of garlic compounds: Allyl cysteine, alliin, allicin, and allyl disulfide. J. Med. Food.

[30] Kaczara P., Sarna T., Burke J.M. (2010). Dynamics of H2O2 availability to ARPE-19 cultures in models of oxidative stress. Free Radic. Biol. Med..

[31] Atanasov A.G., Waltenberger B., Pferschy-Wenzig E.-M., Linder T., Wawrosch C., Uhrin P., Temml V., Wang L., Schwaiger S., Heiss E.H. (2015). Discovery and resupply of pharmacologically active plant-derived natural products: A review. Biotechnol. Adv..

[32] Ncube B., Van Staden J. (2015). Tilting Plant Metabolism for Improved Metabolite Biosynthesis and Enhanced Human Benefit. Molecules.

[33] Shang A., Cao S.-Y., Xu X.-Y., Gan R.-Y., Tang G.-Y., Corke H., Mavumengwana V., Li H.-B. (2019). Bioactive Compounds and Biological Functions of Garlic (*Allium sativum L*.). Foods.

[34] Hattori A., Yamada N., Nishikawa T., Fukuda H., Fujino T. (2005). Antidiabetic effects of ajoene in genetically diabetic KK-A(y) mice. J. Nutr. Sci. Vitaminol..

[35] Wang S.-L., Liu D.E.S., Liang E.-S., Gao Y.-H., Cui Y., Liu Y.-Z., Gao W. (2015). Protective effect of allicin on high glucose/hypoxia-induced aortic endothelial cells via reduction of oxidative stress. Exp. Ther. Med..

[36] Saravanan G., Ponmurugan P. (2010). Beneficial effect of S-allylcysteine (SAC) on blood glucose and pancreatic antioxidant system in streptozotocin diabetic rats. Plant Foods Hum. Nutr..

[37] Ashraf R., Khan R.A., Ashraf I. (2011). Garlic (*Allium sativum*) supplementation with standard antidiabetic agent provides better diabetic control in type 2 diabetes patients. Pak. J. Pharm. Sci..

[38] Perkins B.A., Rabbani N., Weston A., Ficociello L.H., Adaikalakoteswari A., Niewczas M., Warram J., Krolewski A.S., Thornalley P. (2012). Serum levels of advanced glycation endproducts and other markers of protein damage in early diabetic nephropathy in type 1 diabetes. PLoS ONE.

[39] Liu T., Zhang L., Joo D., Sun S.-C. (2017). NF-κB signaling in inflammation. Signal Transduct. Target. Ther..

[40] Zhang C. (2008). The role of inflammatory cytokines in endothelial dysfunction. Basic Res. Cardiol..

[41] Berrone E., Beltramo E., Solimine C., Ape A.U., Porta M. (2006). Regulation of intracellular glucose and polyol pathway by thiamine and benfotiamine in vascular cells cultured in high glucose. J. Biol. Chem..

[42] Rios E.C., Szczesny B., Soriano F.G., Olah G., Szabo C. (2015). Hydrogen sulfide attenuates cytokine production through the modulation of chromatin remodeling. Int. J. Mol. Med..

[43] Melino S., Leo S., Toska Papajani V. (2019). Natural Hydrogen Sulfide Donors from Allium sp. as a Nutraceutical Approach in Type 2 Diabetes Prevention and Therapy. Nutrients.

