# Supplementary material for: Allithiamine Alleviates Hyperglycaemia-Induced Endothelial Dysfunction
Source: Nutrients. 2020 Jun 5;12(6):1690. doi: 10.3390/nu12061690 (PMC7352751; doi:10.3390/nu12061690)
Supplement: Supplementary file 1 [file nutrients-12-01690-s001.pdf]

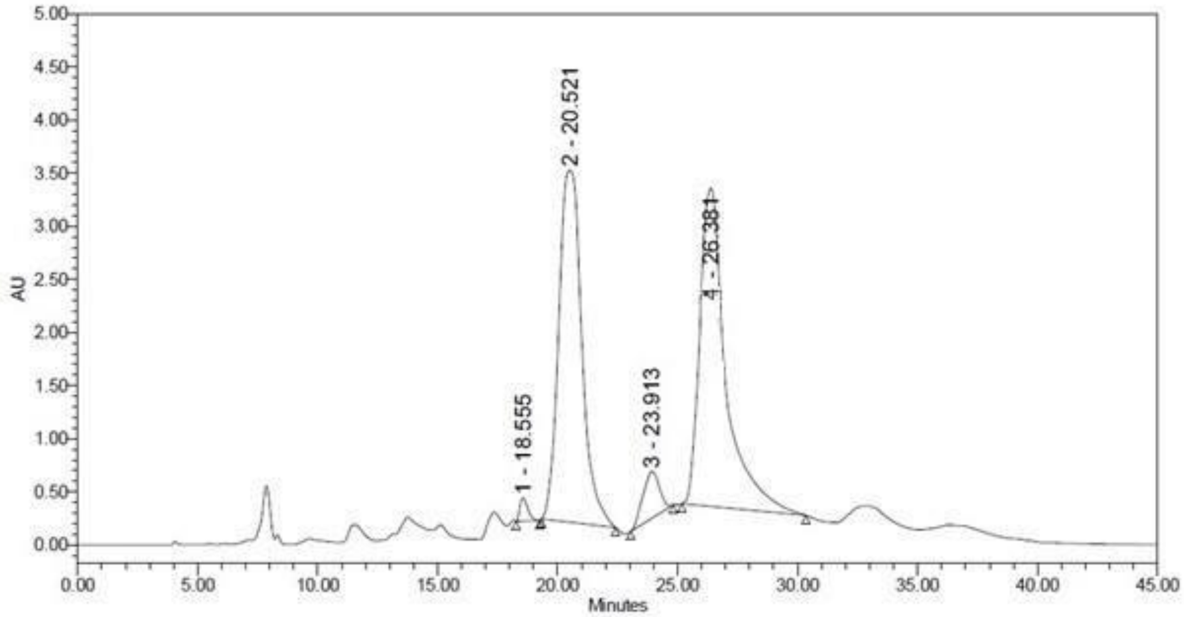

Figure S1. HPLC chromatogram of allithiamine at 250 nm. Allithiamine was eluted at 26.38 min, as we identified with MALDI-TOF and HPLC-MS.

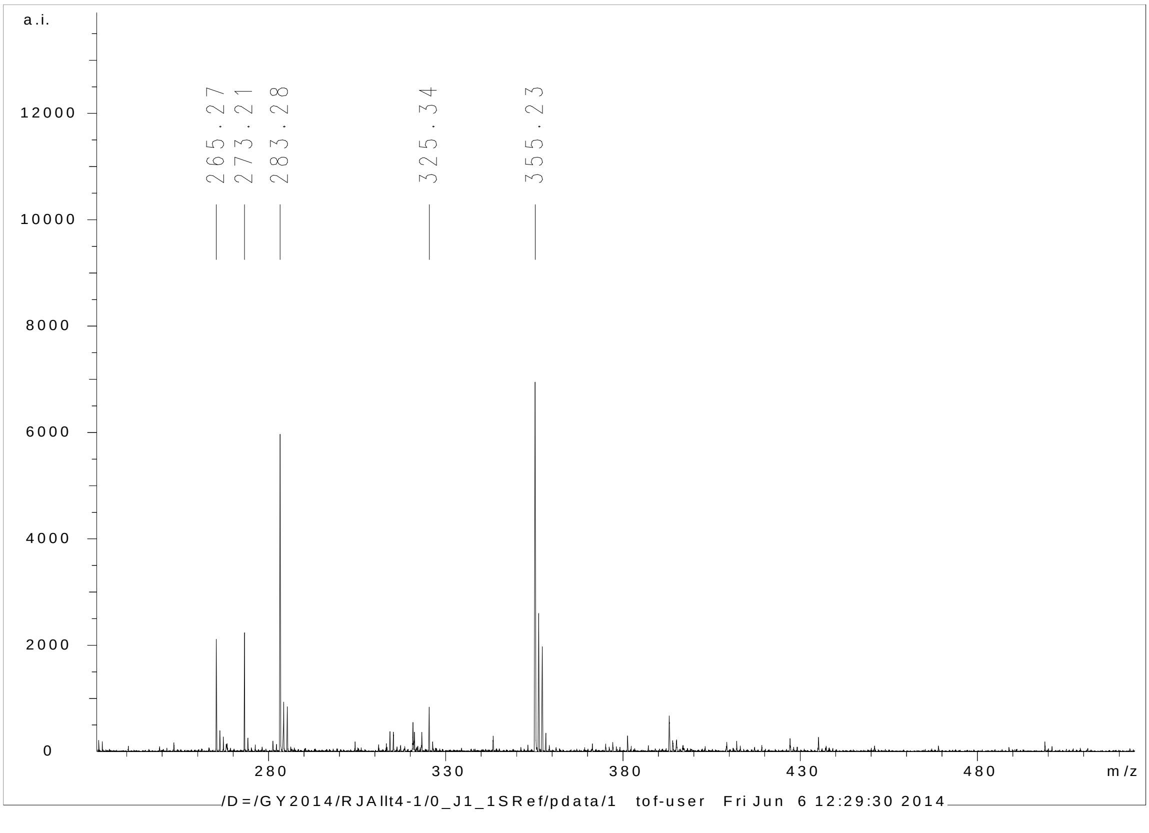

Figure S2. MALDI-TOF spectrum of purified allithiamine.

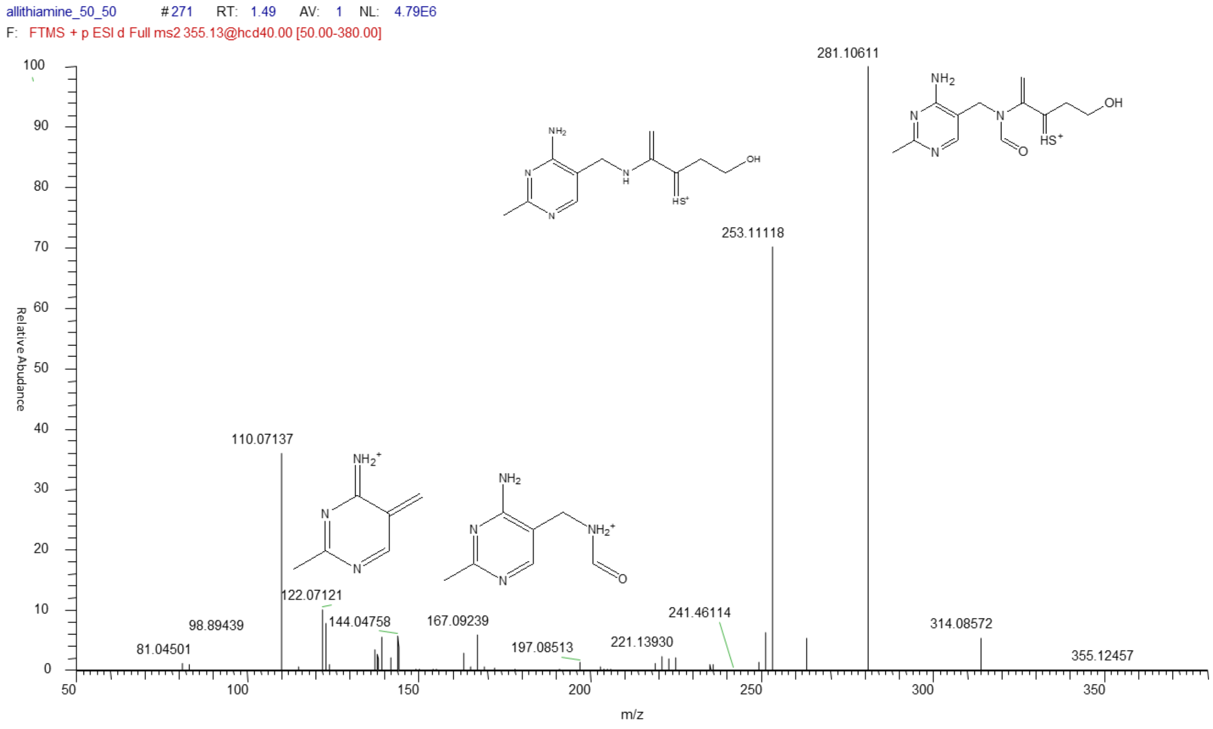

Figure S3. MS^2 spectrum of allithiamine.
